# Maxillary first premolar shape (and not size) as an indicator of sexual dimorphism: A 2D geomorphometric study

**DOI:** 10.12688/f1000research.111382.3

**Published:** 2024-02-16

**Authors:** Srikant Natarajan, Junaid Ahmed, Nidhin Philip Jose, Shravan Shetty

**Affiliations:** 1 Department of Oral Pathology and Microbiology, Manipal College of Dental Sciences Mangalore, Mangalore, Manipal Academy of Higher Education, Manipal, Karnataka, 575001, India; 2 Department of Oral Medicine and Radiology, Manipal College of Dental Sciences Mangalore, Mangalore, Manipal Academy of Higher Education, Manipal, Karnataka, 575001, India; 3 Department of Orthodontics and Dentofacial Orthopaedics, Manipal College of Dental Sciences Mangalore, Mangalore, Manipal Academy of Higher Education, Manipal, Karnataka, 575001, India

**Keywords:** Sexual dimorphism, Geometric morphometry, Procrustes analysis, Maxillary first premolar, Principle component analysis, Tooth form, Tooth shape

## Abstract

**Introduction:**

The primary aim of the study is to evaluate the morphological form of the maxillary first premolar using 2D geomorphometry and evaluate the sexually dimorphic characteristics.

**Methods:**

The present study was carried out on standardized photographs of right Maxillary first premolar from 120 dental casts (60 male and 60 females). Twenty landmarks (based on geometric and anatomic evidence) were marked on the tooth using TPSdig software and analysed using Morpho J applying procrustes analysis and discriminant function analysis.

**Results:**

The results showed similar centroid sizes between gender (p = 0.541). Procrustes ANOVA for shape analysis showed a greater dimorphism between sexs (f value of 1.35; p value=0.0793).  Discriminant function analysis based on the procrustes coordinates showed an overall accuracy of 74.2 % in classifying sex based on the landmark coordinates with correct classification of  48/60 (80.00%) females and 41/60 (68.33) males.

**Conclusion:**

Shape of the tooth can be measured objectively using geometric morphometric methods which can be utilized to identify the sex of an individual. The enamel is derived from ectoderm and once formed does not change during the life. The tooth’s structure and shape are determined by the sex chromosomes, which is well represented as sexual dimorphism. The study evaluates the occlusal and contact area morphology of premolars. These are important parameters considered during restorative treatment, functional rehabilitation and forensic investigations.

## Introduction

In biology, “size” and “shape” are vital to describe an organism or a component of an organism, and expressing these involves the use of morphometry. “Size” is usually represented by linear and angular measurements of an entity. “Shape” on the other hand, is more complex to visualize and involves robust statistical procedures. Shape information is essential for bioarchaeology, anthropology, and forensic sciences to interpret evidence obtained from human remains. Rohlf and Marcus (1993) have reviewed the various procedures utilized in describing shape in biology and have termed the use of geometric morphometric analysis as a revolution in describing the “shape”. ^1^ The geomorphometric analysis involves defining landmarks on the biological structure in two or three dimensions followed by statistical procedures using this data for visualizing the changes in shape. Further, the analysis also graphically represents the landmark variations on transformation grids to identify the deviations seen between species, gender, etc. Forensic anthropologists usually employ these landmark coordinates to define the biological profile. ^2^

Sexual dimorphism in dentition is a well-established feature. Sexual dimorphism in a tooth may be attributed to variations in genetics, epigenetic factors, and the influences of sex hormones.

The shape of the tooth is determined in the “morphodifferentiation stage” of tooth formation which corresponds to the cap and bell stage of odontogenesis. The tooth’s shape is decided by the epithelium’s infolding during the cap stage, which results from the expansion of the predecessor bud stage. Independent of the size of the tooth, deeper or additional infolding may result in the creation of conspicuous ridges, extra cusps, and tubercles. The term “non-metric traits of the tooth” refers to these anatomical characteristics. Non-metric traits of the tooth are essential for the dentists to plan single tooth restorations, as the internal tooth structure varies as per the outer contours. The variations in the tooth shape may also affect development of occlusion by orthodontics and prosthodontists. In their latest work, Chowdhry A *et al.* (2023) assessed 20 non-metric features of human dentition. They report sexual dimorphism in incisor and molar features in their research. Their research revealed that males and females had slightly larger proportions of premolar accessory cusps in first and second premolars, respectively. ^3^ These patterns show the shape of the premolars and are independent of tooth size. The present study explores the change in the shape of the premolars using landmark based morphometric evaluation. Premolars are in the fields of influence of different genes modulating anterior and posterior dentition. These teeth are unique in the permanent dentition as they do not have a deciduous counterpart. Thus, our pilot study focused on the evaluation of sexual dimorphism of the premolar class of the tooth.

The reasons for sexual dimorphism of tooth are different from the craniofacial bones. The facial skeleton including the mandible exhibit sexual dimorphism either directly or indirectly due to the hormones and activity of the muscles attached to them. However tooth are less influenced by hormones and are not affected by the muscle activity directly. ^4^

In a study by Guatelli-Steinberg *et al.* (2008) on seven different populations, they found no significant association of sex hormone concentrations post-birth and tooth patterning. ^5^ However, Ribeiro D *et al* (2013) have demonstrated a significant role of intrauterine testosterone levels in dental development and size. ^6^ Taking cognizance of the varied reports pertaining to hormonal regulation of tooth size/shape, genetic influence on tooth shape must be considered primary. Genes that influence tooth patterning during odontogenesis are located in the sex chromosomes. Genes polymorphisms of MSX1, PAX 9, AXIN2 and EDA are associated with hypodontia and change in tooth morphology. ^7^

Sexual dimorphism has been researched by numerous morphometric studies involving linear measurements of width, length and diagonal measurements of teeth, measurements of areas of the occlusal surfaces, etc. ^8^^–^^10^ Geomorphometric analysis of shape is a relatively new research modality to evaluate shape of the teeth. The maxillary first premolar is a distinctive tooth segment, strategically positioned between the anterior canines, which are responsible for tearing, and the posterior molars, which facilitate chewing. Morphologically, this tooth mirrors a canine when viewed from the buccal aspect and a molar in terms of the expanded surface area observed in the occlusal aspect. Uniquely, no other tooth possesses a hexagonal occlusal outline, making the occlusal surface of this tooth particularly distinctive. It is therefore prudent to study the occlusal surface of the maxillary first premolar. Additionally, these teeth exhibit the least amount of attrition and variation with age, and are known to demonstrate the highest degree of sexual dimorphism. ^11^^–^^14^

The aim of the present present study is to evaluate the geometric morphometric variations of landmarks of the maxillary first premolar as viewed from its occlusal aspect and evaluate its sexual dimorphism.

## Methods

This study was conducted on the Dakshina Kannada population of Karnataka, India. Dakshina Kannada, also known as South Canara, is the southern coastal district of Karnataka State, covering an area of 4859 square kilometres. This district is bordered by the sea to the west, the Western Ghats to the east, Udupi district to the north, and Kerala State to the south. ^15^

The study commenced following the approval by the institutional ethics committee of a dental college in Dakshina Kannada region (vide ref no. 20018, dated 16 ^th^ March 2020). Dental study casts of 120 individuals were retrieved from the archives of Department of Orthodontics, Manipal College of Dental Sciences, Mangalore. Broad written consent was taken from the patients during treatment, for use of the plaster casts for research assuring anonymization. Individuals born and brought up in Dakshina Kannada region were included in the study and their study casts were retrieved. The maxillary pre-treatment dental casts (poured in dental stone) of individuals meeting the inclusion criterion; were retrieved for photography. One of the inclusion criteria for choosing the dental study model was having an undamaged maxillary right first premolar which was free of cavities, wear, restorations, or crown fabrication. The exclusion criterion was any indication of developmental abnormality of the tooth in the subject. Age, sex and demographic details were noted from the patient management system. The randomization of the orthodontics patient box numbers was done using random numbers generated from www.random.org. The study was a time bound study to be completed in three months’ time. Total 120 random number pairs were generated, distributed as 60 Females and 60 Male individuals with an age range of 12–26 years.

Twenty landmarks that are measured in two dimensions were used to analyze the form of the maxillary first premolar. We require 36 samples in each group, taking into account the 4 degrees of freedom lost for the landmark coordinates’ translation, scaling, and rotation. We have taken a sample of 60 in each group to improve the power of the study.

Standardised images of the first maxillary premolar’s occlusal surface were taken with a Canon EOS 700D camera (Canon Inc., Japan) using macro mode. Each cast model was placed in the center of the field of focus of the lens with a ruler placed adjacent to the cast (positioned at the occlusal surface level to avoid magnification error). An intermediate value diaphragm was used for an adequately focused photograph of the premolar’s occlusal surface. The Maxillary first premolar was positioned with the cemento-enamel junction (CEJ) perpendicular to the optical axis making it parallel to the camera lens as suggested by Wood and Abbot. ^16^ The photographs were saved in Tag Image File Format (*.tiff) format for transfer to the landmark marking software “TPSdig” for windows available at https://www.sbmorphometrics.org/soft-dataacq.html.

**Figure 1.**
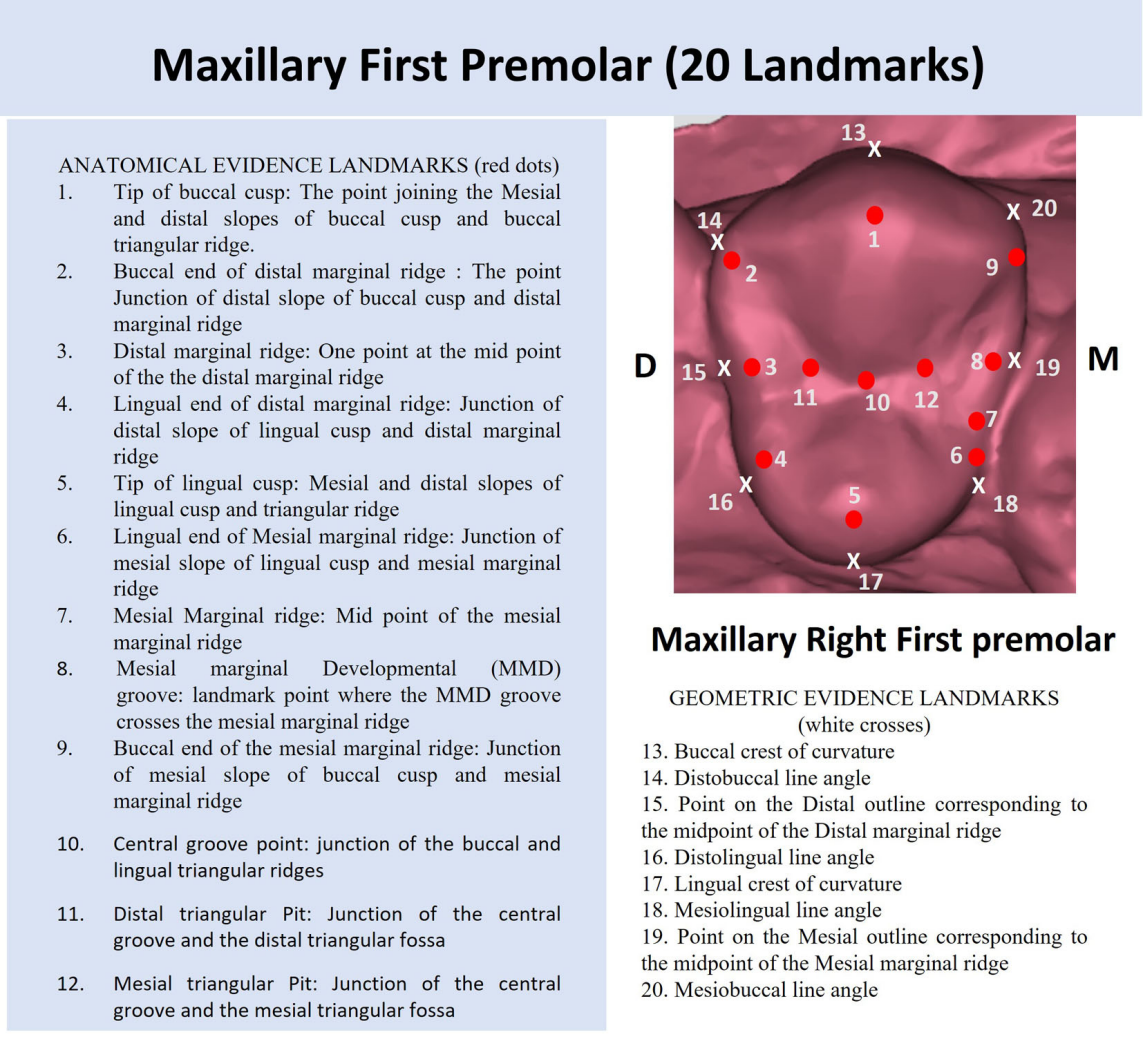
Description of the landmarks based on anatomic and geometric evidence on a right maxillary first premolar.

The landmarks were determined based on the anatomy (anatomic evidence) as well as the geometric contours (geometric evidence) of the tooth. A total of 20 landmarks were identified (12 based on anatomical evidence of cusp, ridges and grooves, and 8 based on geometric evidence of crest of curvature and line angles) as shown in Figure 1.

Using the TPS dig and TPS util software, the landmarks on the premolar were marked as part of the landmark data gathering process.

Using the TPSutil software, the *.tps file was generated of 120 photographs of the maxillary casts in high resolution. This was followed by the landmark acquisition using TPSdig2 software. Using the landmark selection tool, the 20 landmarks (as described in Figure 1) were defined for each maxillary right first premolar of the 120 individuals.

Landmarks of 20 samples (10 male and 10 female) were marked in an interval of 2 weeks by one of the authors (S.N). The sets of coordinates was used to perform the Intraclass correlation coefficient (ICC), to test the reliability of the landmarks. The analysis showed excellent agreement with ICC values of 0.91 with p value of <0.001 indicating high level of interobserver consistency in marking the landmarks.

With great care, the software’s scale was calibrated to match the ruler in every shot. The MorphoJ software (version 1.07a) was used to analyze the obtained landmark coordinates. Procrustes superimposition, principal component analysis and discriminant function were the statistical techniques used and transformation grids and graphical representations were generated.

In brief the process involved scaling of the landmark coordinate data and superimposition using Procrustes technique. Following this a covariance matrix was generated and then principal component analysis (PCA) was performed. Procrustes ANOVA was used to evaluate the differences between the sexes. The classification power of the shape data to classify Sex based on premolar shape was calculated using Discriminant Function Analysis. To make the analysis more robust the threshold of the p value was set at 0.003.

## Results

One hundred and twenty casts of patients included in the study included 60 Females and 60 Males having a mean age of 18.62±2.50 years (males 19.47±1.74 years and Females 17.85±2.81 years).

**Figure 2.**
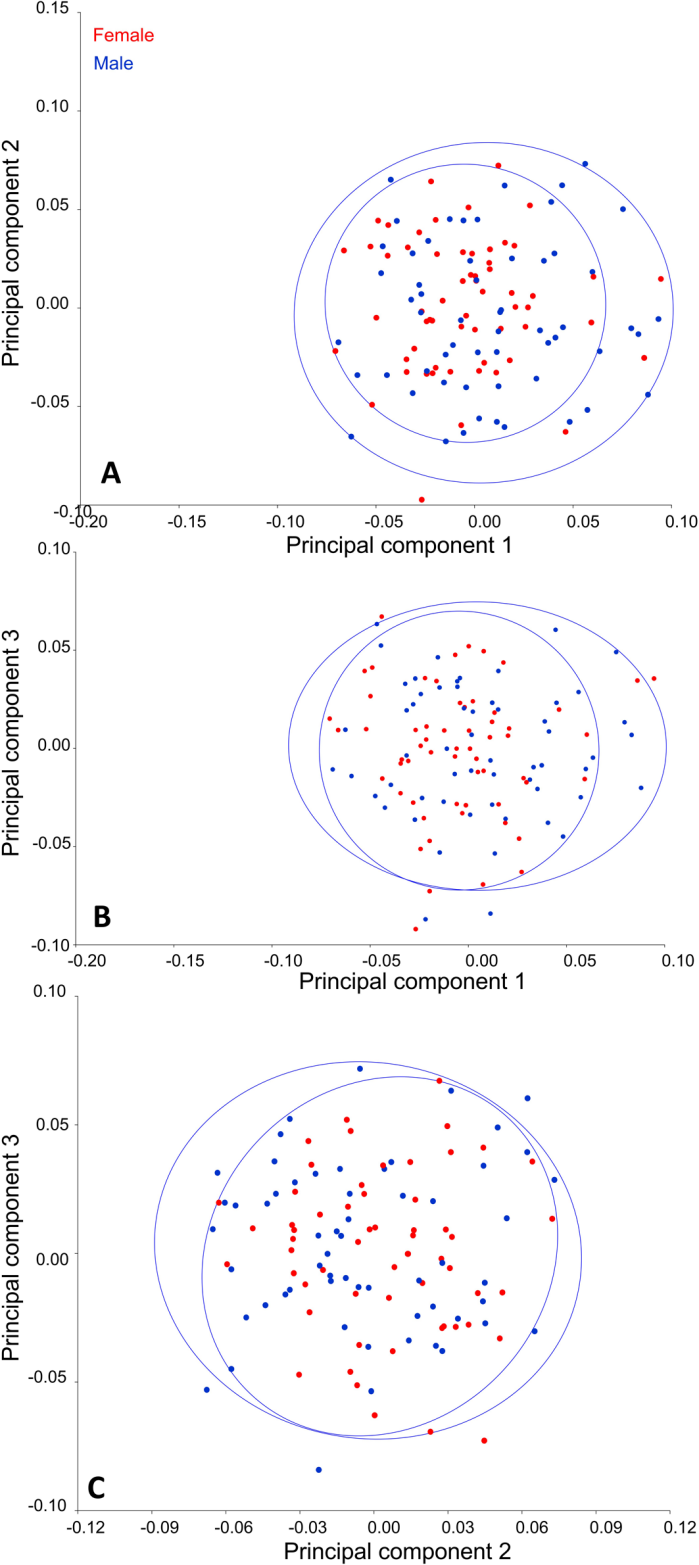
PCA dispersion graph. A=PC1 vs PC2; B=PC1 vs PC3 and C=PC2 vs PC3, Red dots represent female and Blue dots represent males.

Principal Components Analysis (PCA) showed that the first 13 principal components accounted for 80% of the maxillary first premolar variance, with the first five representing 54% of the variability ( Table 1). The scatter was evenly noted on either side of the scatter plot axis, indicating a homogenous distribution of landmarks among individuals. The variability was seen more in males than in females ( Figure 2).

**Table 1.** Eigenvalues and percentage of variance for the maxillary first premolar obtained by Principal Components Analysis.

	Eigenvalues	% Variance	Cumulative %
1	0.00156422	14.446	14.446
2	0.00134636	12.434	26.88
3	0.00112552	10.394	37.274
4	0.00106471	9.833	47.107
5	0.00074739	6.902	54.009
6	0.00052772	4.874	58.882
7	0.00051288	4.737	63.619
8	0.00040629	3.752	67.371
9	0.00039623	3.659	71.03
10	0.00035931	3.318	74.349
11	0.0003174	2.931	77.28
12	0.00026537	2.451	79.731
13	0.00024262	2.241	81.971
14	0.00023835	2.201	84.172
15	0.00021997	2.031	86.204
16	0.00018584	1.716	87.92
17	0.00016198	1.496	89.416
18	0.00012851	1.187	90.603
19	0.00012635	1.167	91.77
20	0.00011211	1.035	92.805
21	0.00010531	0.973	93.778
22	0.00009908	0.915	94.693
23	0.00009307	0.86	95.552
24	0.00007782	0.719	96.271
25	0.00006463	0.597	96.868
26	0.000059	0.545	97.412
27	0.0000556	0.514	97.926
28	0.00004509	0.416	98.342
29	0.00003801	0.351	98.693
30	0.00003301	0.305	98.998
31	0.00002925	0.27	99.268
32	0.00002316	0.214	99.482
33	0.00001948	0.18	99.662
34	0.00001525	0.141	99.803
35	0.00001351	0.125	99.928
36	0.00000782	0.072	100

**Figure 3.**
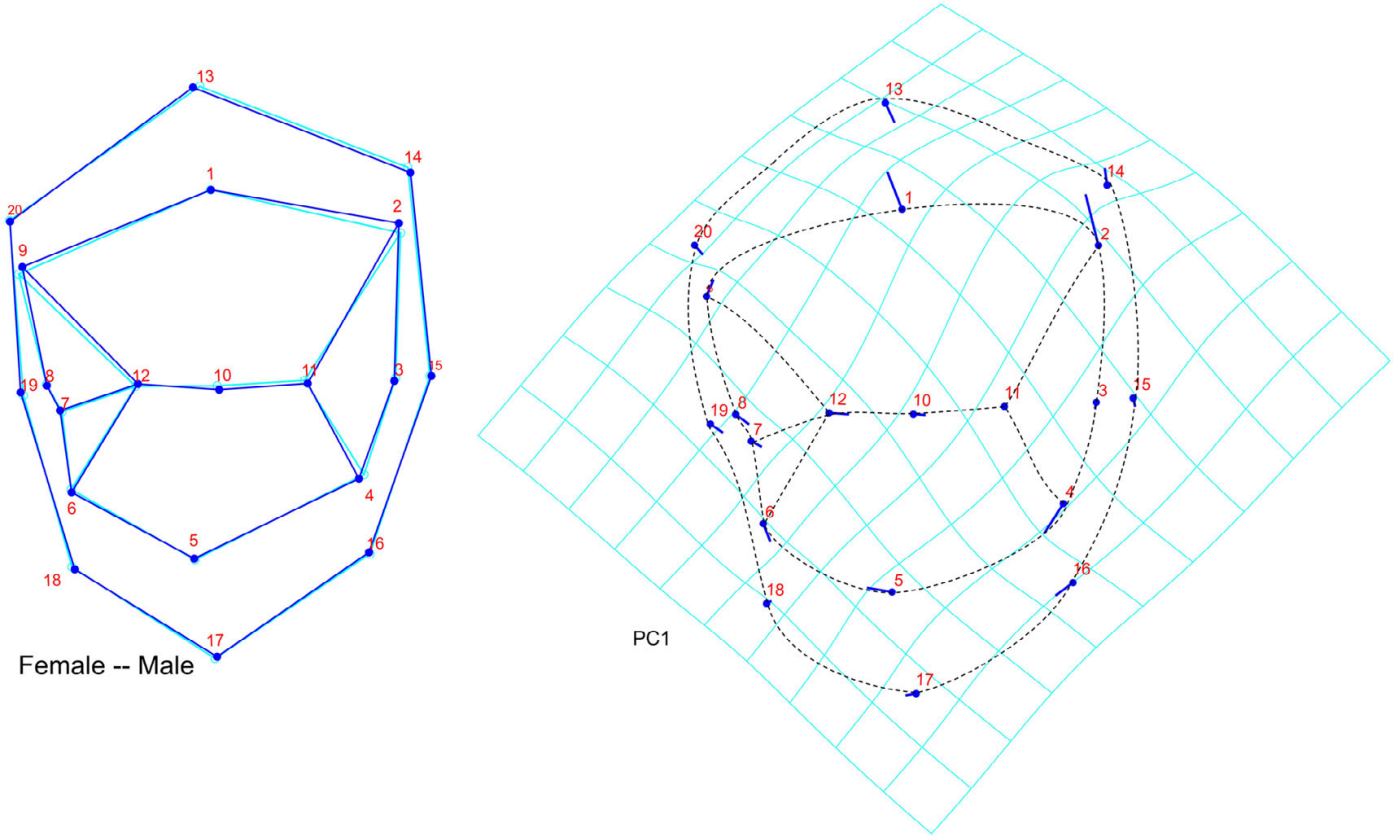
Left: Wireframe graph showing the variation in the landmarks between males and females using Discriminant function analysis Right: Deformation graph showing the variability of the landmarks in the individuals in PC1 (score factor of 0.10); The lollipop graphs show the mean shape of the landmarks as circles and the relative position change of the landmarks is represented by sticks.

The deformation graph showed prominent variability in the lingual direction of the buccal cusp tip and buccal translation of the buccal as well as lingual crest of curvature. Lingual cusp tip has a propensity to move more mesially. The distal end of the distobuccal cusp ridge and the mesial end of the mesiolingual cusp ridge tends to be shifted more towards the middle of the buccolingual dimension. The central groove is relatively standard in position exhibiting minimum buccolingual variation. The mesial marginal developmental groove remains lingual to the groove at all times, but shows some variation buccolingually ( Figure 3).

On comparison of the centroid size, females had a mean centroid size of 14.44±0.73 which was marginally smaller compared to the male individuals’ centroid size of 14.53±0.89 units. This was however not statistically significant with a p value of 0.541 (t=1.98). Procrustes ANOVA for shape analysis showed a greater variation with an f value of 1.35 and p value of 0.0793, indicating an increased variation in shape of the teeth among gender when compared to size. This result indicated that shape of the premolar was showed greater difference compared to the centroid.

**Figure 4.**
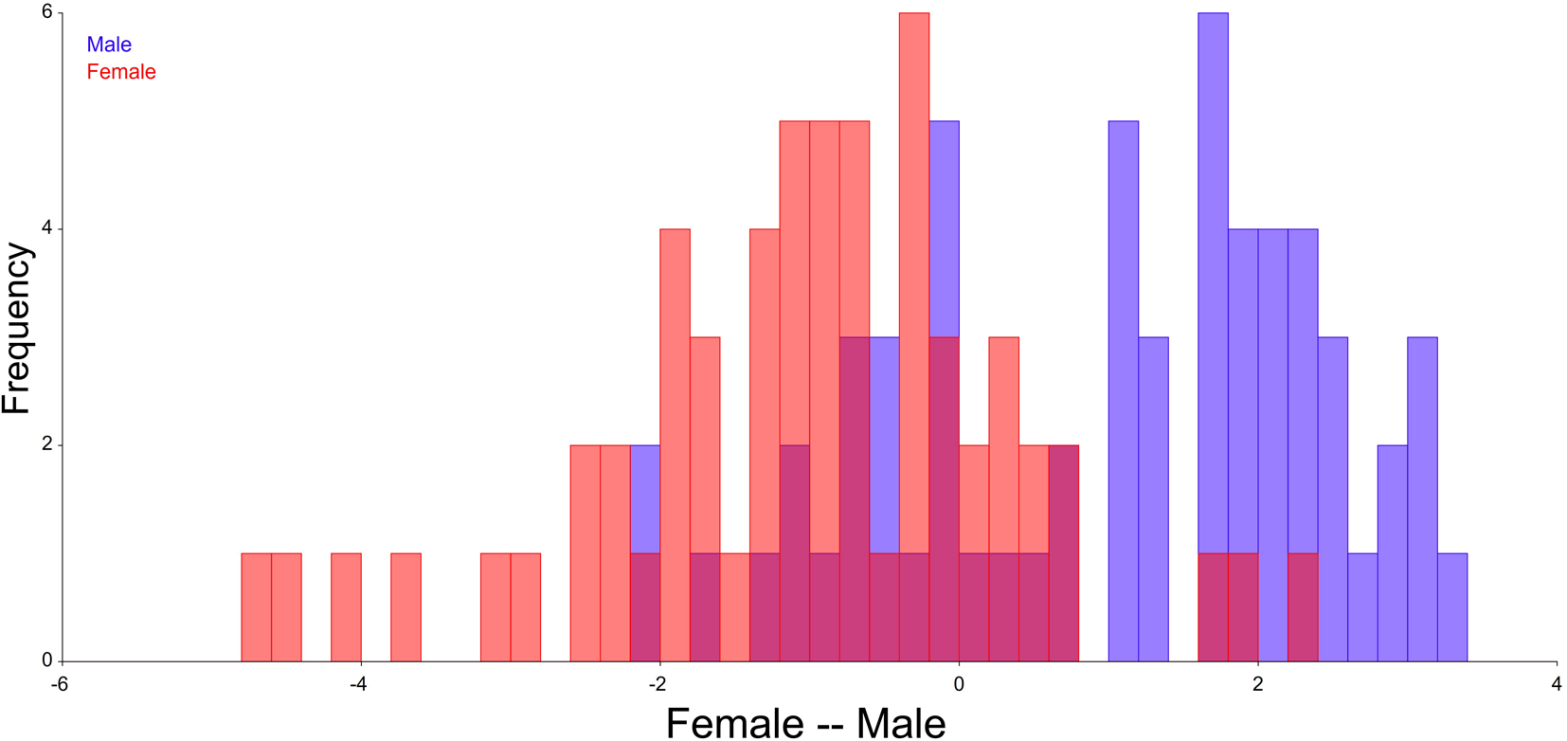
Classification of gender based on the discriminant function analysis.

Discriminant function analysis was performed based on the procrustes coordinates. There was 74.2 percent accuracy in classification of gender based on the landmark coordinates. The accuracy was 48/60 (80.0%) among females and 41/60 (68.33%) among males accounting for 89/120 (74.2%) in the total sample ( Figure 4).

## Discussion

The quantification of an object’s geometric shape by measurement of landmark coordinates is done using geometric morphometric analysis. This method utilizes multivariate statistical procedures that allow preservation of the landmark data in its original geometric shape and enables us to visualize the shape changes in real dimensions. ^17^There are various methods of evaluation of shape or form of a biological structure. These include Euclidean Distance Matrix analysis, ^18^ Elliptical Fournier analysis ^19^ and the most researched and understood procrustes superimposition method. ^20^

Maxillary first premolar is particularly an essential tooth for taxonomic classification. The tooth has a characteristic asymmetry due to the prominent mesial marginal developmental groove and depression making the mesial outline concave compared to distal outline. Bailey and Lynch (2014) have assessed the shape of the mandibular premolars in Neanderthal and Modern humans and found their classification to be more accurate in modern humans with an accuracy of 98.1% as compared to Neanderthals who had an accuracy of 65%. ^19^ The shape of a tooth is said to be a result of genetic drift rather than environmental factors. ^19^ Genes play a primary role in morphodifferentiation of teeth. MSX, DLX, PAX9 genes are responsible for histo- and morpho-differentiation of tooth germ during odontogenesis. Studies have shown that MSX 1 mutation leads to agenesis of teeth especially the premolar segment. ^21^ SPRY2, GAS1 and RUNX2 are potential candidate genes which influence the formation of secondary dentition including premolars. ^22^ These studies indicate that genes play an important role in formation and morphology of premolar.

Only a limited number of studies have been conducted to analyze the shape of premolars using geometric morphometry. In our study, the centroid sizes did not show a significant variation in size of the premolars. This is in line with the other studies in Indian population. Yong *et al.* (2018) have studied the sexual dimorphism of human premolars in the Australian population and found that the centroid size did not show any significant difference by sex. However, Procrustes ANOVA showed significant effects of sex, accounting for 1.1% variation. ^20^ This is in concordance with our present study where we found shape of the tooth to indicate greater sexual dimorphism than the size as seen by procrustes ANOVA. Banerjee A *et al.* (2016) found no significant difference in the odontometric profile of the maxillary first premolar. The male and the female teeth were similar in the mesiodistal, buccolingual dimensions, crown widths and the cervical angulations. ^23^ We did not find significant difference in the shape of the occlusal aspect of the maxillary first premolar. Similar findings are reported by López-Lázaro S *et al.* (2020), where they found significant sexual dimorphism in the second premolar but not in the first premolar. ^24^ Zorba E *et al.* (2011), studied the maxillary postcanine dentition and demonstrated that the maxillary first premolar was the most dimorphic tooth after canine. ^14^

One of the limitations of our study is the 2 dimensional analysis. The buccolingual inclination of the premolar might affect the landmark visualization in a two dimension. This can be overcome by incorporation of the third dimension of the coordinates, and performing a 3D geomorphometric analysis. This would yield a better discriminating ability of the landmarks. Yong R *et al.* (2018) have done an analysis in Australian population using 3D Geometric morphometry and found no significant difference in shape or centroid size in premolars. ^20^ Secondly, a study evaluating the shape variables of all the premolars and molars of the human arch would give an all-inclusive assessment of tooth shape.

For future work, newer mathematical and computational models can be explored for shape analysis of teeth. The newer techniques would be capable in obtaining optimal parameters from the landmark data. In this regard, Choi G *et al.* (2020) in their recent research have compared area based, procrustes based methods with their new shape analysis technique using quasi-conformational theory. They have demonstrated superior results using their newer conformational theory in delineating gender and ancestry among indigenous and European origin Australian population. They have stated that, procrustes based approach gives satisfactory accuracy in discrimination, however, the Teichmuller distance method used is superior owing to the methodologies incorporating mean and Gaussian curvature analysis. ^25^

Potential avenues for expanding the study could also include integrating data on shape and size for the evaluation of sexual dimorphism. The emergence of open-source software modules, such as R and its extensions, could facilitate such an analysis, potentially providing deeper insights into shape of a premolar in the form space.

## Conclusion

2D geomorphometric analysis of the maxillary first premolar was performed utilizing 20 landmarks of geometric and anatomical evidences. The literature shows that size shows minimal variation between gender. ^12^^,^^17^ However, the shape using the 20 landmark coordinate data of the premolar teeth, was able to discriminate gender with an accuracy of 74.2% (as demonstrated by discriminant function analysis). Analysis of the transformation grid and lollipop graphs showed that the maximum variation was in relation to the positioning of the distobuccal cusp ridge end and the distal outline of the buccal surface, both of which are more buccally placed in males. Such variations play an important role in reproduction of the premolar morphology during restoration and tooth alignment. Further, the shape coordinates can be used to estimate sex of the individual as an adjunct in forensic investigations of skeletonized remains.

## Data availability

### Underlying data

Figshare. MAXILLARY Premolar Landmark Data. DOI: https://doi.org/10.6084/m9.figshare.24783015. ^26^

This project contains the following underlying data:

• 2 D data of the landmarks of the 120 maxillary first premolars

Data are available under the terms of the Creative Commons Attribution 4.0 International license (CC-BY 4.0).
