# Supplementary material for: Maxillary first premolar shape (and not size) as an indicator of sexual dimorphism: A 2D geomorphometric study
Source: F1000Res. 2024 Feb 16;11:433. Originally published 2022 Apr 19. [Version 3] doi: 10.12688/f1000research.111382.3 (PMC10933566; doi:10.12688/f1000research.111382.3)
Supplement: Peer review history

# Reviewer report: Reviewer response for version 3

**DOI:** 10.5256/f1000research.161964.r247891

**Published:** 2024-03-12

**Authors:** Choy Ker Woon

**Affiliations:** 1 Department of Anatomy, Faculty of Medicine, Universiti Teknologi MARA, Shah Alam, Malaysia

none

Is the work clearly and accurately presented and does it cite the current literature?

Yes

If applicable, is the statistical analysis and its interpretation appropriate?

Yes

Are all the source data underlying the results available to ensure full reproducibility?

Yes

Is the study design appropriate and is the work technically sound?

Yes

Are the conclusions drawn adequately supported by the results?

Yes

Are sufficient details of methods and analysis provided to allow replication by others?

Partly

Reviewer Expertise:

forensic anthropology, geometric morphometric

I confirm that I have read this submission and believe that I have an appropriate level of expertise to confirm that it is of an acceptable scientific standard.

# Reviewer report: Reviewer response for version 2

**DOI:** 10.5256/f1000research.156991.r221535

**Published:** 2023-11-27

**Authors:** Choy Ker Woon

**Affiliations:** 1 Department of Anatomy, Faculty of Medicine, Universiti Teknologi MARA, Shah Alam, Malaysia

My comment for manuscript titled, Maxillary first premolar shape (and not size) as an indicator of sexual dimorphism: A 2D geomorphometric study is as below 

1. Please provide justification for sample size
2. Provide inclusion and exclusion criteria
3. Compare the result of your study with VARIOUS population in the discussion
4. Include inter and intra observer analysis
5. Justify why use first maxillary premolar’s occlusal surface. Enhance your research gap.

Is the work clearly and accurately presented and does it cite the current literature?

Yes

If applicable, is the statistical analysis and its interpretation appropriate?

Yes

Are all the source data underlying the results available to ensure full reproducibility?

Yes

Is the study design appropriate and is the work technically sound?

Yes

Are the conclusions drawn adequately supported by the results?

Yes

Are sufficient details of methods and analysis provided to allow replication by others?

Partly

Reviewer Expertise:

forensic anthropology, geometric morphometric

I confirm that I have read this submission and believe that I have an appropriate level of expertise to state that I do not consider it to be of an acceptable scientific standard, for reasons outlined above.

# Response

**Published:** 2024-02-05

**Authors:** Srikant Natarajan

**Affiliations:** Manipal College of Dental Sciences Mangalore, India

**Reviewer (Choy Ker Woon) Comment 1:**

1. Please provide justification for sample size

**Reply to comment 1:**

 The justification of the sample size is given in the second paragraph of method as follows

 “Twenty landmarks that are measured in two dimensions are used to analyze the form of the maxillary first premolar. We require 36 samples in each group, taking into account the 4 degrees of freedom lost for the landmark coordinates' translation, scaling, and rotation. We have taken a sample of 60 in each group to improve the power of the study.”

**Reviewer Comment 2:**

1. Provide inclusion and exclusion criteria

**Reply to comment 2:**

 Inclusion and exclusion criterion has been included in the first paragraph of the methodology as follows.

 “One of the inclusion criteria for choosing the dental study model was having an undamaged first premolar on the maxillary right, free of cavities, wear, restorations, or crown implantation. The exclusion criterion was any indication of developmental abnormality of the tooth in the subject.”

**Reviewer Comment 3:**

1. Compare the result of your study with VARIOUS population in the discussion

**Reply to comment 3:**

 Only a limited number of studies have been conducted to analyze the shape of premolars using geometric morphometry. The study by Yong et al. 2018 is one such study. Other studies are odontometric in nature and they have been reviewed and added in the discussion section as follows.

 “Banerjee A et al. (2016) found no significant difference in the odontometric profile of the maxillary first premolar. The male and the female teeth were similar in the mesiodistal, buccolingual dimensions, crown widths and the cervical angulations. (Banerjee et al. 2016)

 We did not find significant difference in the shape of the occlusal aspect of the maxillary first premolar. Similar findings are reported by López-Lázaro S et al. (2020), where they found significant sexual dimorphism in the second premolar but not in the first premolar. (López-Lázaro et al. 2020)

 Zorba E et al. (2011), studied the maxillary postcanine dentition and demonstrated that the maxillary first premolar was the most dimorphic tooth after canine. “

**Reviewer (Choy Ker Woon) Comment 4:**

1. Include inter and intra observer analysis

**Reply to comment 4:**

 We have included the intra observer analysis in the manuscript as follows

 “Landmarks of 20 samples (10 male and 10 female) were marked in an interval of 2 weeks by one of the authors (S.N). The sets of coordinates was used to perform the Intraclass correlation coefficient (ICC), to test the reliability of the landmarks. The analysis showed excellent agreement with ICC values of 0.91 with p value of <0.001 indicating high level of interobserver consistency in marking the landmarks.”

**Reviewer Comment 5:**

1. Justify why use first maxillary premolar’s occlusal surface. Enhance your research gap.

**Reply to comment 5:**

 It is indeed a relevant suggestion as to why we chose the occlusal surface of the premolars for our study. We have incorporated this rationale into the introduction section and have elaborated on the necessity of addressing this research gap.

 “The maxillary first premolar is a distinctive tooth segment, strategically positioned between the anterior canines, which are responsible for tearing, and the posterior molars, which facilitate chewing. Morphologically, this tooth mirrors a canine when viewed from the buccal aspect and a molar in terms of the expanded surface area observed in the occlusal aspect. Uniquely, no other tooth possesses a hexagonal occlusal outline, making the occlusal surface of this tooth particularly distinctive. It is therefore prudent to study the occlusal surface of the maxillary first premolar. Additionally, these teeth exhibit the least amount of attrition and variation with age, and are known to demonstrate the highest degree of sexual dimorphism. (Zorba et al. 2011; Angadi et al. 2013; Sathawane et al. 2020; Bianchi et al. 2023)”

# Reviewer report: Reviewer response for version 2

**DOI:** 10.5256/f1000research.156991.r213191

**Published:** 2023-10-16

**Authors:** Aman Chowdhry

**Affiliations:** 1 Faculty of Dentistry, Jamia Millia Islamia, New Delhi, Delhi, India

The inclusions suggested have been added. the manuscript appears apt to the best of my knowledge.

Is the work clearly and accurately presented and does it cite the current literature?

Partly

If applicable, is the statistical analysis and its interpretation appropriate?

Yes

Are all the source data underlying the results available to ensure full reproducibility?

Yes

Is the study design appropriate and is the work technically sound?

Yes

Are the conclusions drawn adequately supported by the results?

Partly

Are sufficient details of methods and analysis provided to allow replication by others?

Yes

Reviewer Expertise:

Dental anatomy, Dental Anthropology, Forensic odontology, Oral Pathology, reserach methodology, evidence based dentistry.

I confirm that I have read this submission and believe that I have an appropriate level of expertise to confirm that it is of an acceptable scientific standard.

# Reviewer report: Reviewer response for version 1

**DOI:** 10.5256/f1000research.123101.r182639

**Published:** 2023-07-13

**Authors:** Julia Aramendi

**Affiliations:** 1 McDonald Institute for Archaeological Research, University of Cambridge, Cambridge, England, UK

The paper entitled ‘Maxillary first premolar shape (and not size) as an indicator of sexual dimorphism: A 2D geomorphometric study’ presents the study of the maxillary first premolar in a human population of India using a 2D geometric morphometrics approach. The study intends to evaluate the morphological differences between male and female individuals and states that the differences encountered could be linked to genetic-driven factors.

 In my opinion, the methodology applied is adequate; however, I think there are some methodological issues that should be considered by the authors before the paper can be accepted for indexing.

 The most problematic aspect of the study is related to sample size. One of the main concerns when applying statistical tests such as Discriminant Analyses or Canonical Variate Analyses is related to the number of landmarks used with regard to sample size in each group.

 For geometric morphometrics, there is a rule that states that the smallest group within the sample has to exceed the number of variables used in the analysis. In the case of the present study, the number of variables is 34, as it is calculated as the number of landmarks (19 in the present case) multiplied by the number of dimensions (2 as the analysis is performed in the 2-dimensional space), minus the degrees of freedom removed after Procrustes superimposition (in this case, 4 for the rotation, translation and scaling of the landmark coordinates). The number of male and female specimens is lower than the number of variables, as it is equal to 33 and 22, respectively.

 Analyses such as CVA or DFA have long been known to be highly sensitive to the data used as input and prone to exaggerate the separation between samples, even if none is supposed to exist, when the sample size is small or when samples are imbalanced (Albrecht 1992; Mitteroecker and Bookstein 2011; Rohlf 2021; Courtenay 2023).

 Comparing the results provided in the present study, it seems that differences between the female and male groups might have been exaggerated, as a result of sample size, since the PCAs show a high overlapping degree between both samples. Thus, in order to verify the difference in shape observed by the authors, it would be necessary to enlarge the sample size.

 On top of that, there are other methodological problems that should be considered by the authors;

1. The figure explaining the landmarks is not clear enough and sensitivity tests regarding landmarking accuracy should be performed to make sure that the analyses can be reproduced. The intra and interobserver’s error could be considered by repeatedly landmarking at least some of the individuals to then assess if landmark definitions are robust enough. I say that because according to Figure 2 there seems to be an outlier, that might be explained by landmarking errors.
2. The methodology should be explained in greater detail. There is no mention to a Procrustes superimposition, step that is necessary to perform geometric morphometrics, neither do the authors explain how the analyses they use function (PCA, DFA, CVA, ANOVA). I am afraid that the lack of explanation might be linked to some of the methodological problems of the study.
3. I also think that results are not explained properly and that unnecessary information is provided instead (i.e., Table 1). Additionally, the threshold for p values to detect significant difference between samples should be set at 0.003, as it is more robust against Type I statistical error, by lowering the risk of making a Type I error from 28.9% when p = 0.05 to 4.5% (Courtenay et al., 2021)

In general, it seems that there are some concepts of the methodological approach that have not been fully understood. Explanations are sometimes too vague and/or inaccurate (e.g., “Shape” on the other hand, is more complex to visualize and involves robust statistical procedures).

 I am also worried about the data collection process as many researchers do not scale the landmarks properly after using software pieces such as TPSdig2. If landmark data were not scaled after collection, the analysis on centroid size might also be misleading. For that reason, it would be great to have access to the raw landmark data gathered from the 55 individuals included in the study.

 It would also be interesting if the authors could combine the shape and size data and perform analyses in form space. Unfortunately, those types of studies cannot be performed in MorphoJ, so a different software piece should be used instead (e.g., R).

 All things considered, I think the study has the potential to be of interest to the scientific community, if the methodological concerns raised here are tackled. Unfortunately, at the moment, I do not think that the outcomes can be used to support their conclusions, since there are some fundamental problems in their methodological approach.

Is the work clearly and accurately presented and does it cite the current literature?

Partly

If applicable, is the statistical analysis and its interpretation appropriate?

No

Are all the source data underlying the results available to ensure full reproducibility?

No

Is the study design appropriate and is the work technically sound?

Partly

Are the conclusions drawn adequately supported by the results?

No

Are sufficient details of methods and analysis provided to allow replication by others?

Partly

Reviewer Expertise:

Geometric morphometrics; human evolution; biological anthropology; biomechanics

I confirm that I have read this submission and believe that I have an appropriate level of expertise to state that I do not consider it to be of an acceptable scientific standard, for reasons outlined above.

## References

[1] : Assessing the affinities of fossils using canonical variates and generalized distances. *Human Evolution* .1992;7(4) : 10.1007/BF02436412 49-69 10.1007/BF02436412

[2] : Linear Discrimination, Ordination, and the Visualization of Selection Gradients in Modern Morphometrics. *Evolutionary Biology* .2011;38(1) : 10.1007/s11692-011-9109-8 100-114 10.1007/s11692-011-9109-8

[3] : Why Clusters and Other Patterns Can Seem to be Found in Analyses of High-Dimensional Data. *Evolutionary Biology* .2021;48(1) : 10.1007/s11692-020-09518-6 1-16 10.1007/s11692-020-09518-6

[4] : Can we Restore Balance to Geometric Morphometrics? A Theoretical Evaluation of how Sample Imbalance Conditions Ordination and Classification. *Evolutionary Biology* .2023;50(1) : 10.1007/s11692-022-09590-0 90-110 10.1007/s11692-022-09590-0

[5] : Hyperspectral imaging and robust statistics in non-melanoma skin cancer analysis. *Biomed Opt Express* .2021;12(8) : 10.1364/BOE.428143 5107-5127 10.1364/BOE.428143 34513245 PMC8407807

# Response

**Published:** 2024-02-05

**Authors:** Srikant Natarajan

**Affiliations:** Manipal College of Dental Sciences Mangalore, India

**Reviewer Comment 1:**

 In my opinion, the methodology applied is adequate; however, I think there are some methodological issues that should be considered by the authors before the paper can be accepted for indexing.

 The most problematic aspect of the study is related to sample size. One of the main concerns when applying statistical tests such as Discriminant Analyses or Canonical Variate Analyses is related to the number of landmarks used with regard to sample size in each group.

 For geometric morphometrics, there is a rule that states that the smallest group within the sample has to exceed the number of variables used in the analysis. In the case of the present study, the number of variables is 34, as it is calculated as the number of landmarks (19 in the present case) multiplied by the number of dimensions (2 as the analysis is performed in the 2-dimensional space), minus the degrees of freedom removed after Procrustes superimposition (in this case, 4 for the rotation, translation and scaling of the landmark coordinates). The number of male and female specimens is lower than the number of variables, as it is equal to 33 and 22, respectively.

**Reply to comment 1:**

 Thank you for the suggestions. We have repeated the analysis with **an increased sample size** and **in** the methods we have included one more landmark in the middle of the mesial marginal ridge, in the mesial aspect of the premolar occlusal outline accounting for 20 landmarks in total. The sample size calculation mainly depends on the dimensions used for the landmarks and the number of transformations. In our study accounting for the two dimensions used for landmarks and removal of 4 degrees of freedom we need 36 samples per group. As per the suggestion of the reviewer we have substantially increased the sample size to 60 in each group to make it total of 120 samples.

**Reviewer Comment 2:**

 Analyses such as CVA or DFA have long been known to be highly sensitive to the data used as input and prone to exaggerate the separation between samples, even if none is supposed to exist, when the sample size is small or when samples are imbalanced (Albrecht 1992; Mitteroecker and Bookstein 2011; Rohlf 2021; Courtenay 2023). Comparing the results provided in the present study, it seems that differences between the female and male groups might have been exaggerated, as a result of sample size, since the PCAs show a high overlapping degree between both samples. Thus, in order to verify the difference in shape observed by the authors, it would be necessary to enlarge the sample size.

**Reply to comment 2:**

 We have expanded the sample size for our study and conducted the analysis again, ensuring a 1:1 male to female sample ratio with 60 samples each. This adjustment has effectively addressed the previous imbalance and mitigated the overemphasis of differences that can occur in studies with smaller sample sizes.

**Reviewer Comment 3:**

 On top of that, there are other methodological problems that should be considered by the authors;

 The figure explaining the landmarks is not clear enough and sensitivity tests regarding landmarking accuracy should be performed to make sure that the analyses can be reproduced. The intra and interobserver’s error could be considered by repeatedly landmarking at least some of the individuals to then assess if landmark definitions are robust enough. I say that because according to Figure 2 there seems to be an outlier, that might be explained by landmarking errors.

**Redoing the analysis will change the graphs also - please see the graph linked here.**

**Reply to comment 3:**

 During the reiteration of the analysis with an expanded sample size, we employed the Intraclass Correlation Coefficient to assess the consistency of the landmark coordinates within a subset of 20 samples. The results of this evaluation have been incorporated into the manuscript as follows

 “Landmarks of 20 samples (10 male and 10 female) were marked in an interval of 2 weeks by one of the authors (S.N). The sets of coordinates was used to perform the Intraclass correlation coefficient (ICC), to test the reliability of the landmarks. The analysis showed excellent agreement with ICC values of 0.91 with p value of <0.001 indicating high level of interobserver consistency in marking the landmarks.”

 Also the graphs and figures have been redone as the analysis is repeated with increased samples.

**Reviewer Comment 4:**

 The methodology should be explained in greater detail. There is no mention to a Procrustes superimposition, step that is necessary to perform geometric morphometrics, neither do the authors explain how the analyses they use function (PCA, DFA, CVA, ANOVA). I am afraid that the lack of explanation might be linked to some of the methodological problems of the study.

**Reply to comment 4:**

 We have provided a comprehensive explanation of the sequence of Procrustes superimposition, the generation of a covariance matrix, and the execution of PCA, Procrustes ANOVA, and discriminant function analysis. We sincerely appreciate your attention to this matter and trust that our detailed explanation will assist future researchers in accurately reproducing these methods.

**Reviewer (Julia Aramendi) Comment 5:**

 I also think that results are not explained properly and that unnecessary information is provided instead (i.e., Table 1). Additionally, the threshold for p values to detect significant difference between samples should be set at 0.003, as it is more robust against Type I statistical error, by lowering the risk of making a Type I error from 28.9% when p = 0.05 to 4.5% (Courtenay et al., 2021)

**Reply to comment 5:**

 Following your advice and the provided references, we have adjusted the p-value threshold to 0.003. We have found that neither the shape **nor** the size of the tooth is statistically significant.

**Reviewer Comment 6:**

 In general, it seems that there are some concepts of the methodological approach that have not been fully understood. Explanations are sometimes too vague and/or inaccurate (e.g., “Shape” on the other hand, is more complex to visualize and involves robust statistical procedures). I am also worried about the data collection process as many researchers do not scale the landmarks properly after using software pieces such as TPSdig2. If landmark data were not scaled after collection, the analysis on centroid size might also be misleading. For that reason, it would be great to have access to the raw landmark data gathered from the 55 individuals included in the study.

**Reply to comment 6:**

 Having expanded our sample size, we have submitted a new dataset of 120 premolar landmarks for review, accessible via a link in the manuscript. We would like to emphasize that the landmarks identified were scaled. The photographs were captured with a scale, which was documented alongside the landmarks. This methodology is detailed in the manuscript’s methods section

**Reviewer Comment 7:**

 It would also be interesting if the authors could combine the shape and size data and perform analyses in form space. Unfortunately, those types of studies cannot be performed in MorphoJ, so a different software piece should be used instead (e.g., R).

**Reply to comment 7:**

 We have positively received this suggestion and decided to implement it in a three-dimensional geometric morphometry analysis. This approach may necessitate collaboration with a team proficient in other software, such as ‘R’. We have acknowledged this suggestion and included the absence of this analysis as a limitation of our study in the conclusion.

 “Potential avenues for expanding the study could include integrating data on shape and size for the evaluation of sexual dimorphism. The emergence of open-source software modules, such as R and its extensions, could facilitate such an analysis, potentially providing deeper insights into shape of a premolar in the form space.”

# Reviewer report: Reviewer response for version 1

**DOI:** 10.5256/f1000research.123101.r164380

**Published:** 2023-04-21

**Authors:** Aman Chowdhry

**Affiliations:** 1 Faculty of Dentistry, Jamia Millia Islamia, New Delhi, Delhi, India

- Non metric traits (i.e. shape and not size) should be highlighted clearly with its applications not only in domain of forensic but also other dental specialties (in Introduction).
- Only hormonal influence on shape has been discussed and not specific 1-2 genes or embryology (cap stage) which leads to shaping of particular tooth (in introduction). Later on discussion has genes mentioned but hormones absent. I suggest both should be present in 1 place (discussion / introduction)

- Why premolars was choice of sample to be studied and its implications should be mentioned in manuscript.
- Try and add https://doi.org/10.1186/s41935-023-00329-2 for premolar recent catalogue of trait. Specially for premolar accessory cusp in 24 (PAC-4), premolar accessory cusp in 25 (PAC-5).  
- Please make the methodology/manuscript anonymous (without mentioning the name of particular institute (your details will come affiliation section).
- What is Dakshina Kannada region/population .. please support it with facts/references.
- Mean age of patients/cast included should results, although age range of the included sample can be mentioned in methodology.
- Abbreviations like *.tiff and TPSdig should be either explained or elaborated as acronyms.  
- 3d geomorphometric surface analysis should be mentioned with reference as future prospects

Is the work clearly and accurately presented and does it cite the current literature?

Partly

If applicable, is the statistical analysis and its interpretation appropriate?

Yes

Are all the source data underlying the results available to ensure full reproducibility?

Yes

Is the study design appropriate and is the work technically sound?

Yes

Are the conclusions drawn adequately supported by the results?

Partly

Are sufficient details of methods and analysis provided to allow replication by others?

Yes

Reviewer Expertise:

Dental anatomy, Dental Anthropology, Forensic odontology, Oral Pathology, reserach methodology, evidence based dentistry.

I confirm that I have read this submission and believe that I have an appropriate level of expertise to confirm that it is of an acceptable scientific standard, however I have significant reservations, as outlined above.

## References

[1] : Study of twenty non-metric dental crown traits using ASUDAS system in NCR (India) population. *Egyptian Journal of Forensic Sciences* .2023;13(1) : 10.1186/s41935-023-00329-2 10.1186/s41935-023-00329-2

# Response

**Published:** 2023-10-02

**Authors:** Srikant Natarajan

**Affiliations:** Manipal College of Dental Sciences Mangalore, India

Point 1 

 Non metric traits (i.e. shape and not size) should be highlighted clearly with its applications not only in domain of forensic but also other dental specialties (in Introduction).

 reply 

 A paragraph in the introduction is added defining and highlighting the importance of non metric traits in dentistry

 “The shape of the tooth is determined in the "morphogenetic stage" of tooth formation. The tooth's shape is decided by the epithelium's infolding during the cap stage, which results from the expansion of the predecessor bud stage. Independent of the size of the tooth, deeper or additional infolding may result in the creation of conspicuous ridges, extra cusps, and tubercles. The term "non-metric traits of the tooth" refers to these anatomical characteristics. Non-metric traits of the tooth are essential for the dentists to plan single tooth restorations, as the internal tooth structure varies as per the outer contours. The variations in the tooth shape may also affect development of occlusion by orthodontics and prosthodontists.”

 Point 2 

 Only hormonal influence on shape has been discussed and not specific 1-2 genes or embryology (cap stage) which leads to shaping of particular tooth (in introduction). Later on discussion has genes mentioned but hormones absent. I suggest both should be present in 1 place (discussion / introduction)

 reply 

 As suggested, hormonal and genetic concepts are introduced in the introduction

 “The reasons for sexual dimorphism of tooth are different from the craniofacial bones. The facial skeleton including the mandible exhibit sexual dimorphism either directly or indirectly due to the hormones and their muscle activity. However tooth are less influenced by hormones. (Oettlé et al., 2009)

 In a study by Guatelli-Steinberg *et al.* (2008) on seven different populations, they found no significant association of sex hormone concentrations post-birth and tooth patterning. ^3 ^However, Ribeiro D *et al* (2013) have demonstrated a significant role of intrauterine testosterone levels in dental development and size. ^4 ^Taking cognizance of the varied reports pertaining to hormonal regulation of tooth size/shape, genetic influence on tooth shape must be considered primary. Genes that influence tooth patterning during odontogenesis are located in the sex chromosomes. Genes polymorphisms of MSX1, PAX 9, AXIN2 and EDA are associated with hypodontia and change in tooth morphology. (Kerekes-Máthé et al., 2023)”

 Point 3

 Why premolars was choice of sample to be studied and its implications should be mentioned in manuscript. Try and add https://doi.org/10.1186/s41935-023-00329-2 for premolar recent catalogue of trait. Specially for premolar accessory cusp in 24 (PAC-4), premolar accessory cusp in 25 (PAC-5). 

 Reply 

 The justification of assessing the premolars are explained in the introduction.

 “In their latest work, Chowdhry A et al. (2023) assessed 20 non-metric features of human dentition. They report sexual dimorphism in incisor and molar features in their research.  Their research revealed that males and females had slightly larger proportions of premolar accessory cusps in first and second premolars, respectively. (Chowdhry et al., 2023) These patterns show the shape of the premolars and are independent of tooth size. The present study explores the change in the shape of the premolars using landmark based morphometric evaluation. Premolars are in the fields of influence different genes modulating anterior and posterior dentition. The tooth are unique in the permanent dentition as they do not have a deciduous counter part. Thus, our pilot study focused on the evaluation of sexual dimorphism of the premolar class of the tooth.”

 Point 4

 Please make the methodology/manuscript anonymous (without mentioning the name of particular institute (your details will come affiliation section).

 Reply 

 The methodology is anonymized by removing the name of the institute.

 Point 5

 What is Dakshina Kannada region/population .. please support it with facts/references.

 reply 

 The details of the region is described in the beginning of the methods with an addition of a new reference

 “This study was conducted on the Dakshina Kannada population of Karnataka, India. Dakshina Kannada, also known as South Canara, is the southern coastal district of Karnataka State, covering an area of 4859 square kilometers. This district is bordered by the sea to the west, the Western Ghats to the east, Udupi district to the north, and Kerala State to the south. (Lokesh et al., 2022)

 “

 Point 6

 Mean age of patients/cast included should results, although age range of the included sample can be mentioned in methodology.

 reply 

 The age range is mentioned in the methodology and the mean age is shifted to the results.

 Point 7

 Abbreviations like *.tiff and TPSdig should be either explained or elaborated as acronyms. 

 Reply 

 Explanation is included in the methodology section

 “The photographs were saved in Tag Image File Format (*.tiff) format for transfer to the landmark marking software “TPSdig” for windows available at https://www.sbmorphometrics.org/soft-dataacq.html.”

 Point 8

 3d geomorphometric surface analysis should be mentioned with reference as future prospects

 Reply 

 Yong R et al’s study in 2018 has been quoted and added under the head of future prospects.
